# The genome sequence of the Bee Wolf, *Philanthus triangulum* (Fabricius, 1775) (Hymenoptera: Crabronidae)

**DOI:** 10.12688/wellcomeopenres.24951.1

**Published:** 2025-10-03

**Authors:** Ryan Mitchell, James du Preez, Natural History Museum Genome Acquisition Lab, Darwin Tree of Life Barcoding Collective, Wellcome Sanger Institute Tree of Life Management, Samples and Laboratory team, Wellcome Sanger Institute Scientific Operations: Sequencing Operations, Wellcome Sanger Institute Tree of Life Core Informatics team, Tree of Life Core Informatics collective, Darwin Tree of Life Consortium

**Affiliations:** 1 Independent researcher, Sligo, County Sligo, Ireland; 2 Wellcome Sanger Institute, Hinxton, England, UK

**Keywords:** Philanthus triangulum; Bee Wolf; genome sequence; chromosomal; Hymenoptera

## Abstract

We present a genome assembly from an individual female *Philanthus triangulum* (Bee Wolf; Arthropoda; Insecta; Hymenoptera; Crabronidae). The genome sequence has a total length of 575.71 megabases. The assembly is partially (49.26%) scaffolded into 13 chromosomal pseudomolecules, with the remainder consisting of repetitive sequence that could not be assembled. The mitochondrial genome has also been assembled, with a length of 24.54 kilobases. This assembly was generated as part of the Darwin Tree of Life project, which produces reference genomes for eukaryotic species found in Britain and Ireland.

## Species taxonomy

Eukaryota; Opisthokonta; Metazoa; Eumetazoa; Bilateria; Protostomia; Ecdysozoa; Panarthropoda; Arthropoda; Mandibulata; Pancrustacea; Hexapoda; Insecta; Dicondylia; Pterygota; Neoptera; Endopterygota; Hymenoptera; Apocrita; Aculeata; Apoidea; Crabronidae; Philanthinae; Philanthini; Philanthina; *Philanthus*; *Philanthus triangulum* (Fabricius, 1775) (NCBI:txid280486)

## Background

*Philanthus triangulum* (Fabricius, 1775), commonly known as the European Beewolf, is a solitary digger wasp and predator of the honey bee ( *Apis melifera*). The adult Beewolf, typically measuring 8–17 mm in length, has a black head, thorax, and a yellow abdomen with varied black triangular markings ( Lomholdt, 1984). Males exhibit a distinct pale trident marking on the forehead, while females have a V-shaped mark ( Simon-Thomas & Simon-Thomas, 1980). The wasp has a wide distribution, spanning the Afrotropics and western Palaearctic ( GBIF Secretariat, 2023; Simon-Thomas & Simon-Thomas, 1980).

In Britain, *P. triangulum* has been listed as “vulnerable” ( Falk, 1991; Shirt, 1987). While an official review of this status is required, the European Beewolf does not appear on the more recent UK Biodiversity Action Plan priority species list ( Joint Nature Conservation Committee (JNCC), 2007). Although historically scarce, the wasp is now commonly found throughout the southern counties, extending to the northern midlands with territories set to expand due to climate change ( Bantock, 2010; Olszewski *et al.*, 2022).

Adults are herbivorous and survive on nectar and pollen, while predation of the honey bee serves as a food source for larvae. Females hunt, paralyse and store their prey in caches of up to 6 bees for larvae to feed on ( Lomholdt, 1984). Nests are built in sun-exposed sandy areas including dunes or lowland heaths. Burrows of up to 1 metre in length are excavated over three days using powerful front legs, followed by adjacent side tunnels, terminating in brooding chambers. In colder regions such as England, Beewolves are univoltine; and overwinter, while in warmer climates they may have two broods a year ( Bantock, 2010; Lomholdt, 1984).

The humid microenvironment of brood cells threatens fungal infestation during development. Beewolves possess several fascinating adaptations to mitigate this ( Strohm & Linsenmair, 2001). Female Beewolves preserve food stores by applying a hydrophobic hydrocarbon-based secretion from the post-pharyngeal gland to impair fungal spoilage ( Herzner & Strohm, 2007; Strohm *et al.*, 2008). Furthermore, female adults have been shown to innoculate the brood cell and cocoon with a protective antibiotic-producing actinomycete symbiont, cultured in specialised antennal glands ( Kaltenpoth *et al.*, 2005; Kroiss *et al.*, 2010). Moreover, eggs possess antimicrobial adaptations by producing concentrated nitric oxide to sterilise the brood cell ( Strohm *et al.*, 2019). Such adaptations work synergistically to reduce brood mortality. The genome sequence will help further elucidate its place within Hymenoptera, allowing comparative analysis of the Beewolf’s unique traits and their evolution, the potential to help inform pollinator health research and provide insights into evolutionary mutualisms ( Branstetter *et al.*, 2018).

**Figure 1.**
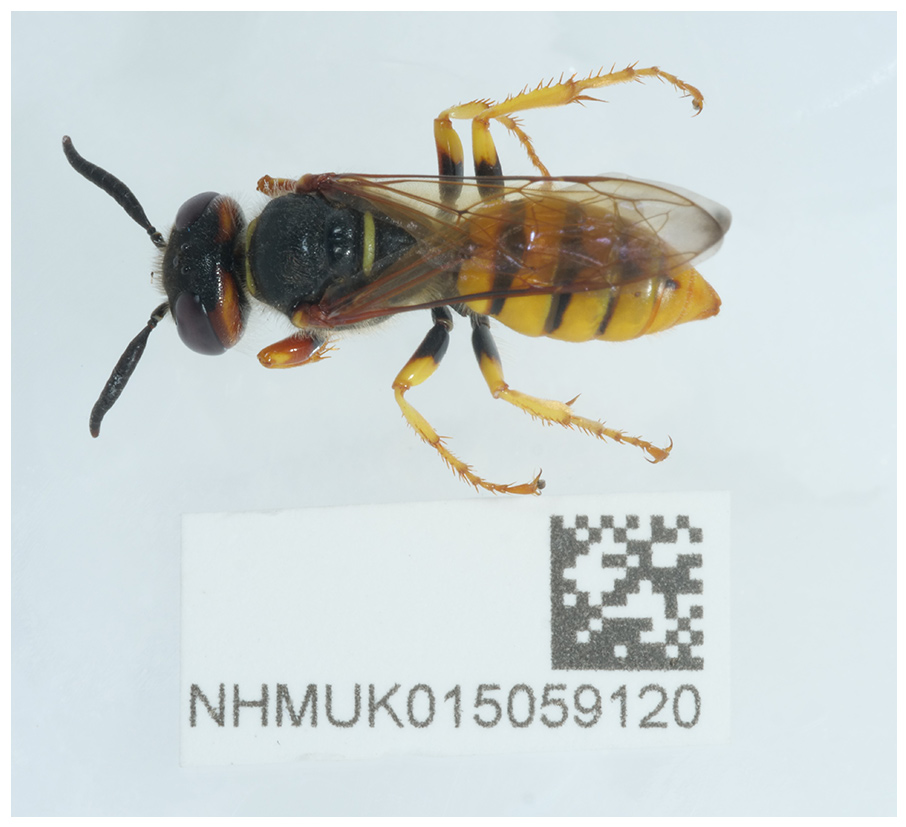
Photograph of the *Philanthus triangulum* (iyPhiTria1) specimen used for genome sequencing.

The assembly was produced using the Tree of Life pipeline from a specimen collected in Buxton Heath, England, United Kingdom ( Figure 1), as part of the Darwin Tree of Life project. It is the first genome for the genus *Philanthus* and one of 14 genomes available for the family Crabronidae as of September 2025 (data obtained via NCBI datasets, O’Leary *et al.*, 2024).

## Methods

### Sample acquisition and DNA barcoding

The specimen used for genome sequencing was an adult female *Philanthus triangulum* (specimen ID NHMUK015059120, ToLID iyPhiTria1; Figure 1), collected from Buxton Heath, England, United Kingdom (latitude 52.75, longitude 1.22) on 2022-07-04. The specimen was collected and identified by Ryan Mitchell (independent researcher). The same specimen was used for RNA sequencing. Sample metadata were collected in line with the Darwin Tree of Life project standards described by Lawniczak *et al*. (2022).

The initial identification was verified by an additional DNA barcoding process according to the framework developed by Twyford *et al.* (2024). A small sample was dissected from the specimen and stored in ethanol, while the remaining parts were shipped on dry ice to the Wellcome Sanger Institute (WSI) (see the protocol). The tissue was lysed, the COI marker region was amplified by PCR, and amplicons were sequenced and compared to the BOLD database, confirming the species identification ( Crowley *et al.*, 2023). Following whole genome sequence generation, the relevant DNA barcode region was also used alongside the initial barcoding data for sample tracking at the WSI ( Twyford *et al.*, 2024). The standard operating procedures for Darwin Tree of Life barcoding are available on protocols.io.

### Nucleic acid extraction

Protocols for high molecular weight (HMW) DNA extraction developed at the Wellcome Sanger Institute (WSI) Tree of Life Core Laboratory are available on protocols.io ( Howard *et al.*, 2025). The iyPhiTria1 sample was weighed and triaged to determine the appropriate extraction protocol. Tissue from the whole organism was homogenised by powermashing using a PowerMasher II tissue disruptor.

HMW DNA was extracted in the WSI Scientific Operations core using the Automated MagAttract v2 protocol. DNA was sheared into an average fragment size of 12–20 kb following the Megaruptor®3 for LI PacBio protocol. Sheared DNA was purified by manual SPRI (solid-phase reversible immobilisation). The concentration of the sheared and purified DNA was assessed using a Nanodrop spectrophotometer and Qubit Fluorometer using the Qubit dsDNA High Sensitivity Assay kit. Fragment size distribution was evaluated by running the sample on the FemtoPulse system. For this sample, the final post-shearing DNA had a Qubit concentration of 25.63 ng/μL and a yield of 1 178.98 ng, with a fragment size of 14.2 kb. The 260/280 spectrophotometric ratio was 1.9, and the 260/230 ratio was 2.05.

RNA was extracted from whole organism tissue of iyPhiTria1 in the Tree of Life Laboratory at the WSI using the RNA Extraction: Automated MagMax™ *mir*Vana protocol. The RNA concentration was assessed using a Nanodrop spectrophotometer and a Qubit Fluorometer using the Qubit RNA Broad-Range Assay kit. Analysis of the integrity of the RNA was done using the Agilent RNA 6000 Pico Kit and Eukaryotic Total RNA assay.

### PacBio HiFi library preparation and sequencing

Library preparation and sequencing were performed at the WSI Scientific Operations core. Libraries were prepared using the SMRTbell Prep Kit 3.0 (Pacific Biosciences, California, USA), following the manufacturer’s instructions. The kit includes reagents for end repair/A-tailing, adapter ligation, post-ligation SMRTbell bead clean-up, and nuclease treatment. Size selection and clean-up were performed using diluted AMPure PB beads (Pacific Biosciences). DNA concentration was quantified using a Qubit Fluorometer v4.0 (ThermoFisher Scientific) and the Qubit 1X dsDNA HS assay kit. Final library fragment size was assessed with the Agilent Femto Pulse Automated Pulsed Field CE Instrument (Agilent Technologies) using the gDNA 55 kb BAC analysis kit.

The sample was sequenced using the Sequel IIe system (Pacific Biosciences, California, USA). The concentration of the library loaded onto the Sequel IIe was in the range 40–135 pM. The SMRT link software, a PacBio web-based end-to-end workflow manager, was used to set-up and monitor the run, and to perform primary and secondary analysis of the data upon completion.

### Hi-C

***Sample preparation and crosslinking***

The Hi-C sample was prepared from 20–50 mg of frozen whole organism tissue of the iyPhiTria1 sample using the Arima-HiC v2 kit (Arima Genomics). Following the manufacturer’s instructions, tissue was fixed and DNA crosslinked using TC buffer to a final formaldehyde concentration of 2%. The tissue was homogenised using the Diagnocine Power Masher-II. Crosslinked DNA was digested with a restriction enzyme master mix, biotinylated, and ligated. Clean-up was performed with SPRISelect beads before library preparation. DNA concentration was measured with the Qubit Fluorometer (Thermo Fisher Scientific) and Qubit HS Assay Kit. The biotinylation percentage was estimated using the Arima-HiC v2 QC beads.

***Hi-C library preparation and sequencing***

Biotinylated DNA constructs were fragmented using a Covaris E220 sonicator and size selected to 400–600 bp using SPRISelect beads. DNA was enriched with Arima-HiC v2 kit Enrichment beads. End repair, A-tailing, and adapter ligation were carried out with the NEBNext Ultra II DNA Library Prep Kit (New England Biolabs), following a modified protocol where library preparation occurs while DNA remains bound to the Enrichment beads. Library amplification was performed using KAPA HiFi HotStart mix and a custom Unique Dual Index (UDI) barcode set (Integrated DNA Technologies). Depending on sample concentration and biotinylation percentage determined at the crosslinking stage, libraries were amplified with 10–16 PCR cycles. Post-PCR clean-up was performed with SPRISelect beads. Libraries were quantified using the AccuClear Ultra High Sensitivity dsDNA Standards Assay Kit (Biotium) and a FLUOstar Omega plate reader (BMG Labtech).

Prior to sequencing, libraries were normalised to 10 ng/μL. Normalised libraries were quantified again and equimolar and/or weighted 2.8 nM pools. Pool concentrations were checked using the Agilent 4200 TapeStation (Agilent) with High Sensitivity D500 reagents before sequencing. Sequencing was performed using paired-end 150 bp reads on the Illumina NovaSeq 6000.

### RNA library preparation and sequencing

Libraries were prepared using the NEBNext ^®^ Ultra™ II Directional RNA Library Prep Kit for Illumina (New England Biolabs), following the manufacturer’s instructions. Poly(A) mRNA in the total RNA solution was isolated using oligo(dT) beads, converted to cDNA, and uniquely indexed; 14 PCR cycles were performed. Libraries were size-selected to produce fragments between 100–300 bp. Libraries were quantified, normalised, pooled to a final concentration of 2.8 nM, and diluted to 150 pM for loading. Sequencing was carried out on the Illumina NovaSeq X to generate 150-bp paired-end reads.

### Genome assembly

Prior to assembly of the PacBio HiFi reads, a database of *k*-mer counts ( *k* = 31) was generated from the filtered reads using FastK. GenomeScope2 ( Ranallo-Benavidez *et al.*, 2020) was used to analyse the *k*-mer frequency distributions, providing estimates of genome size, heterozygosity, and repeat content.

The HiFi reads were assembled using Hifiasm ( Cheng *et al.*, 2021) with the --primary option. Haplotypic duplications were identified and removed using purge_dups ( Guan *et al.*, 2020). The Hi-C reads ( Rao *et al.*, 2014) were mapped to the primary contigs using bwa-mem2 ( Vasimuddin *et al.*, 2019), and the contigs were scaffolded in YaHS ( Zhou *et al.*, 2023) with the --break option for handling potential misassemblies. The scaffolded assemblies were evaluated using Gfastats ( Formenti *et al.*, 2022), BUSCO ( Manni *et al.*, 2021) and MERQURY.FK ( Rhie *et al.*, 2020).

The mitochondrial genome was assembled using MitoHiFi ( Uliano-Silva *et al.*, 2023), which runs MitoFinder ( Allio *et al.*, 2020) and uses these annotations to select the final mitochondrial contig and to ensure the general quality of the sequence.

### Assembly curation

The assembly was decontaminated using the Assembly Screen for Cobionts and Contaminants ( ASCC) pipeline. TreeVal was used to generate the flat files and maps for use in curation. Manual curation was conducted primarily in PretextView and HiGlass ( Kerpedjiev *et al.*, 2018). Scaffolds were visually inspected and corrected as described by Howe *et al*. (2021). Manual corrections included 16 breaks and 19 joins. The curation process is documented at https://gitlab.com/wtsi-grit/rapid-curation. PretextSnapshot was used to generate a Hi-C contact map of the final assembly.

### Assembly quality assessment

The Merqury.FK tool ( Rhie *et al.*, 2020) was run in a Singularity container ( Kurtzer *et al.*, 2017) to evaluate *k*-mer completeness and assembly quality for the primary and alternate haplotypes using the *k*-mer databases ( *k* = 31) computed prior to genome assembly. The analysis outputs included assembly QV scores and completeness statistics.

The genome was analysed using the BlobToolKit pipeline, a Nextflow implementation of the earlier Snakemake version ( Challis *et al.*, 2020). The pipeline aligns PacBio reads using minimap2 ( Li, 2018) and SAMtools ( Danecek *et al.*, 2021) to generate coverage tracks. It runs BUSCO ( Manni *et al.*, 2021) using lineages identified from the NCBI Taxonomy ( Schoch *et al.*, 2020). For the three domain-level lineages, BUSCO genes are aligned to the UniProt Reference Proteomes database ( Bateman *et al.*, 2023) using DIAMOND blastp ( Buchfink *et al.*, 2021). The genome is divided into chunks based on the density of BUSCO genes from the closest taxonomic lineage, and each chunk is aligned to the UniProt Reference Proteomes database with DIAMOND blastx. Sequences without hits are chunked using seqtk and aligned to the NT database with blastn ( Altschul *et al.*, 1990). The BlobToolKit suite consolidates all outputs into a blobdir for visualisation. The BlobToolKit pipeline was developed using nf-core tooling ( Ewels *et al.*, 2020) and MultiQC ( Ewels *et al.*, 2016), with containerisation through Docker ( Merkel, 2014) and Singularity ( Kurtzer *et al.*, 2017).

## Genome sequence report

### Sequence data

**Figure 2.**
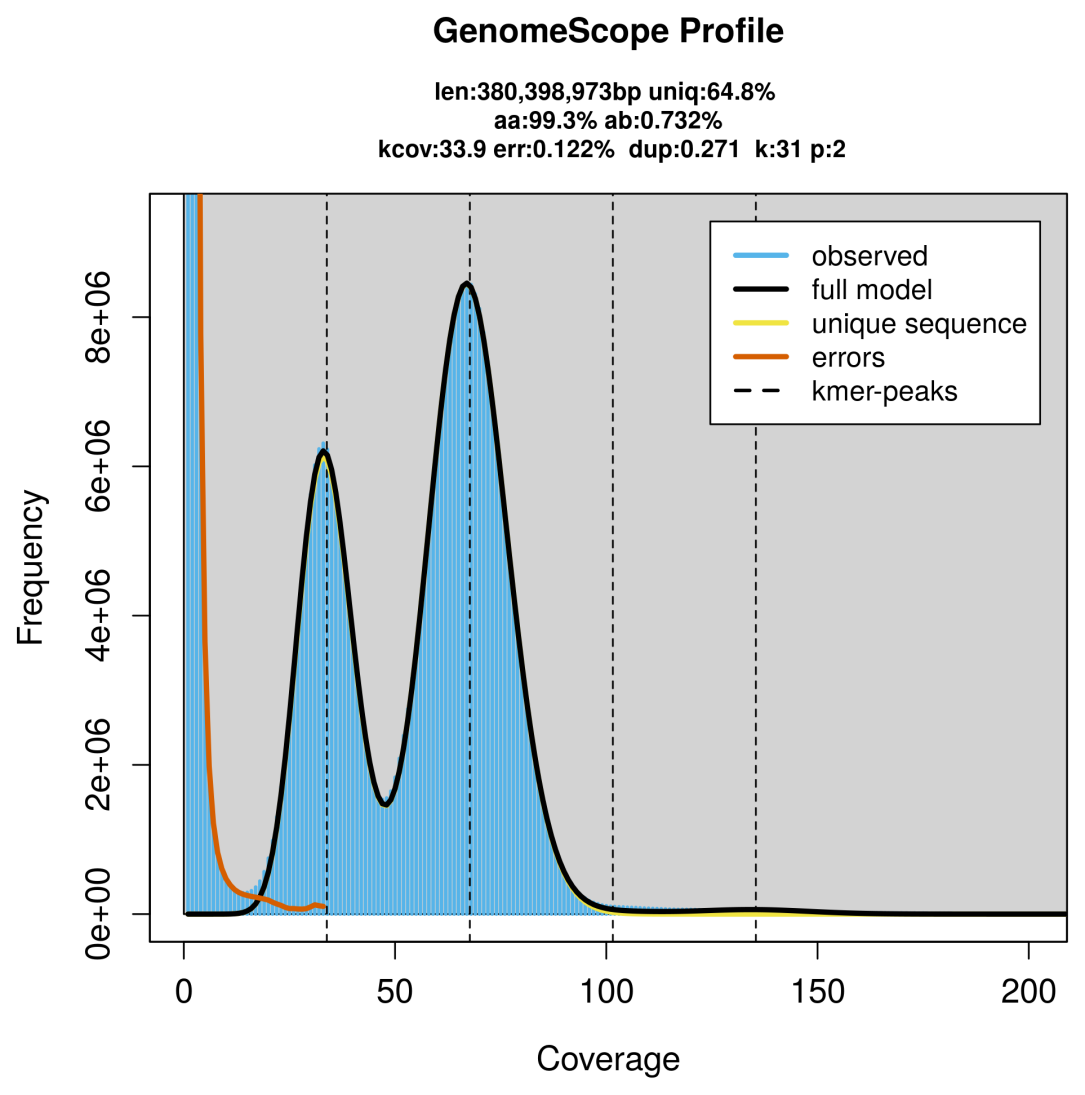
Frequency distribution of *k*-mers generated using GenomeScope2. The plot shows observed and modelled *k*-mer spectra, providing estimates of genome size, heterozygosity, and repeat content based on unassembled sequencing reads.

PacBio sequencing of the *Philanthus triangulum* specimen generated 26.82 Gb (gigabases) from 2.30 million reads, which were used to assemble the genome. GenomeScope2.0 analysis estimated the haploid genome size at 380.40 Mb, with a heterozygosity of 0.73% and repeat content of 35.27% ( Figure 2). These estimates guided expectations for the assembly. Based on the estimated genome size, the sequencing data provided approximately 68× coverage. Hi-C sequencing produced 125.07 Gb from 828.28 million reads, which were used to scaffold the assembly. RNA sequencing data were also generated and are available in public sequence repositories. Table 1 summarises the specimen and sequencing details.

**Table 1.** Specimen and sequencing data for BioProject PRJEB66736.

Platform	PacBio HiFi	Hi-C	RNA-seq
**ToLID**	iyPhiTria1	iyPhiTria1	iyPhiTria1
**Specimen ID**	NHMUK015059120	NHMUK015059120	NHMUK015059120
**BioSample (source individual)**	SAMEA112964339	SAMEA112964339	SAMEA112964339
**BioSample (tissue)**	SAMEA112975504	SAMEA112975504	SAMEA112975504
**Tissue**	whole organism	whole organism	whole organism
**Instrument**	Sequel IIe	Illumina NovaSeq 6000	Illumina NovaSeq X
**Run accessions**	ERR12102449	ERR12102408	ERR15140880
**Read count total**	2.30 million	828.28 million	132.11 million
**Base count total**	26.82 Gb	125.07 Gb	19.95 Gb

### Assembly statistics

The primary haplotype was assembled, and contigs corresponding to an alternate haplotype were also deposited in INSDC databases. The final assembly has a total length of 575.71 Mb in 379 scaffolds, with 118 gaps, and a scaffold N50 of 12.49 Mb ( Table 2).

**Table 2.** Genome assembly statistics.

**Assembly name**	iyPhiTria1.1
**Assembly accession**	GCA_965213405.1
**Alternate haplotype accession**	GCA_965213425.1
**Assembly level**	chromosome
**Span (Mb)**	575.71
**Number of chromosomes**	13
**Number of contigs**	497
**Contig N50**	2.85 Mb
**Number of scaffolds**	379
**Scaffold N50**	12.49 Mb
**Sex chromosomes**	N/A
**Organelles**	Mitochondrion: 24.54 kb

**Figure 3.**
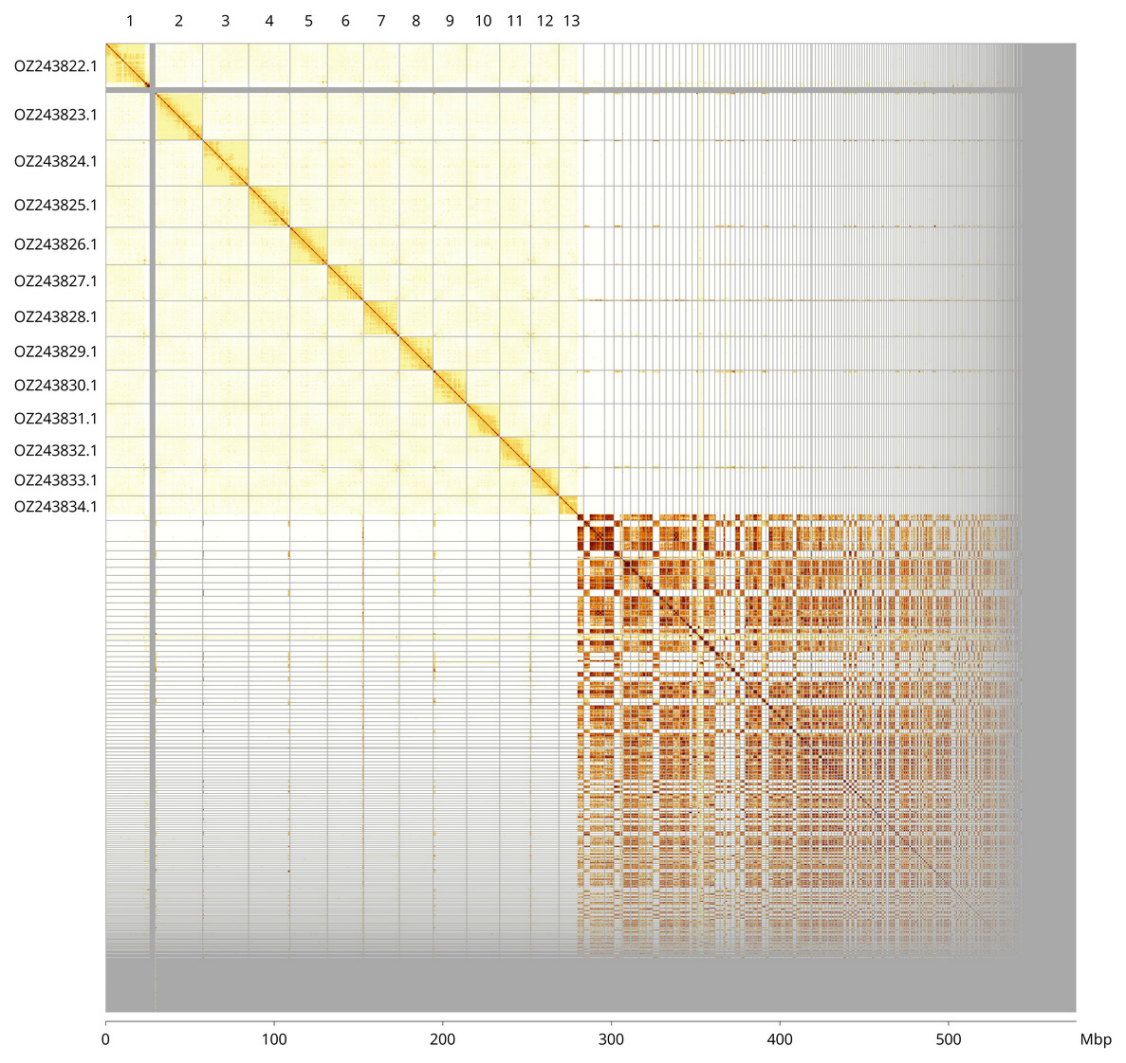
Hi-C contact map of the *Philanthus triangulum* genome assembly. Assembled chromosomes are shown in order of size and labelled along the axes, with a megabase scale shown below. The plot was generated using PretextSnapshot.

The assembly sequence is partially (49.26%) assigned to 13 chromosomal-level scaffolds. These chromosome-level scaffolds, confirmed by Hi-C data, are named according to size ( Figure 3; Table 3). The assembly contains a large number of repetitive sequences that remain fragmented and unplaced.

**Table 3.** Chromosomal pseudomolecules in the primary genome assembly of *Philanthus triangulum* iyPhiTria1.

INSDC accession	Molecule	Length (Mb)	GC%
OZ243822.1	1	29.32	39.50
OZ243823.1	2	28.30	38
OZ243824.1	3	27.24	38
OZ243825.1	4	24.56	38.50
OZ243826.1	5	22.28	38
OZ243827.1	6	21.39	38.50
OZ243828.1	7	21.14	38.50
OZ243829.1	8	20.11	37
OZ243830.1	9	19.87	38.50
OZ243831.1	10	19.51	37
OZ243832.1	11	18.50	38.50
OZ243833.1	12	16.75	38
OZ243834.1	13	14.60	39.50

The mitochondrial genome was also assembled. This sequence is included as a contig in the multifasta file of the genome submission and as a standalone record.

**Figure 4.**
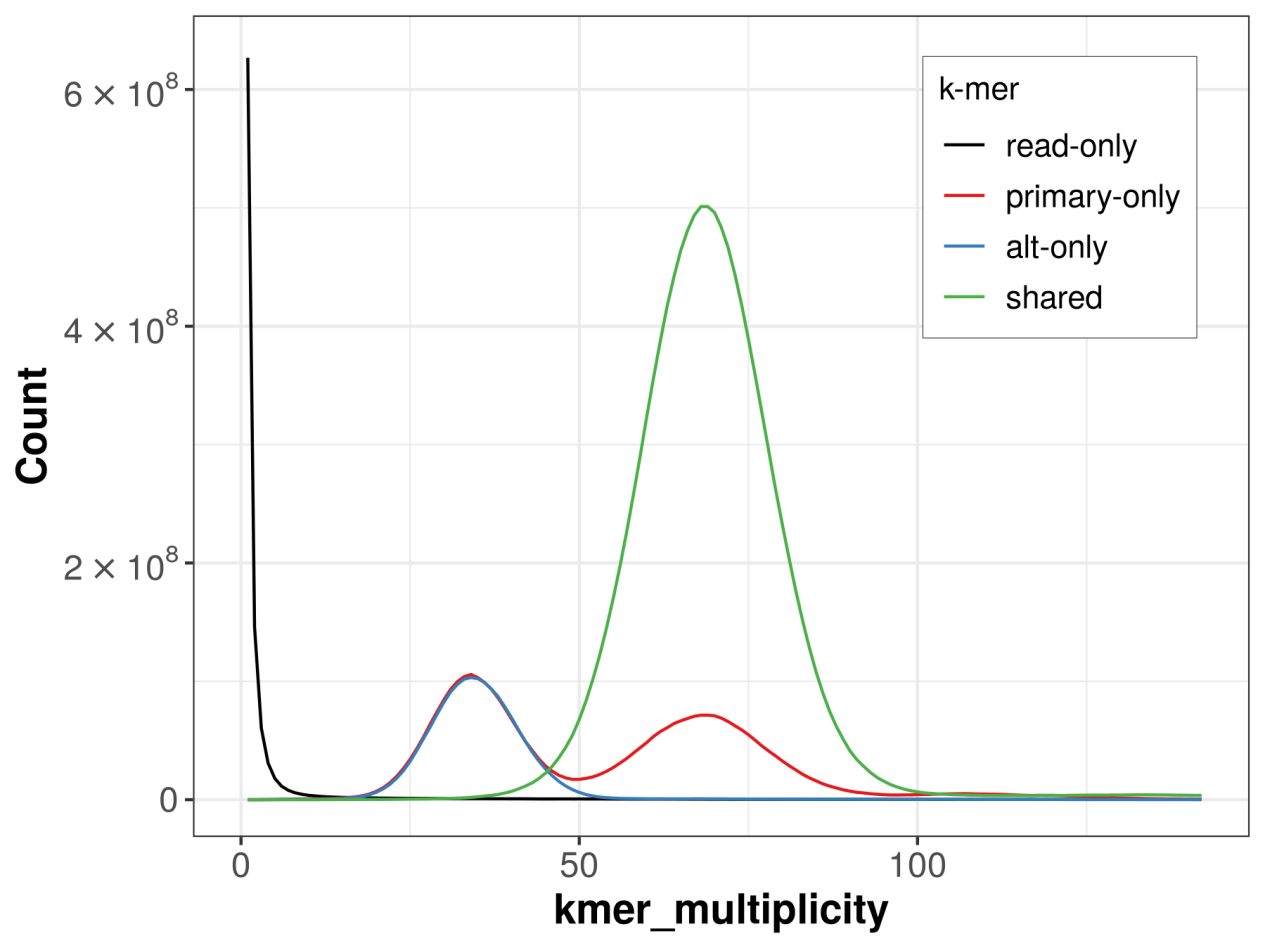
Evaluation of *k*-mer completeness using MerquryFK. This plot illustrates the recovery of *k*-mers from the original read data in the final assemblies. The horizontal axis represents *k*-mer multiplicity, and the vertical axis shows the number of *k*-mers. The black curve represents *k*-mers that appear in the reads but are not assembled. The green curve corresponds to *k*-mers shared by both haplotypes, and the red and blue curves show *k*-mers found only in one of the haplotypes.

The combined primary and alternate assemblies achieve an estimated QV of 65.2. The *k*-mer completeness is 83.31% for the primary assembly, 74.39% for the alternate haplotype, and 99.39% for the combined assemblies ( Figure 4).

**Figure 5.**
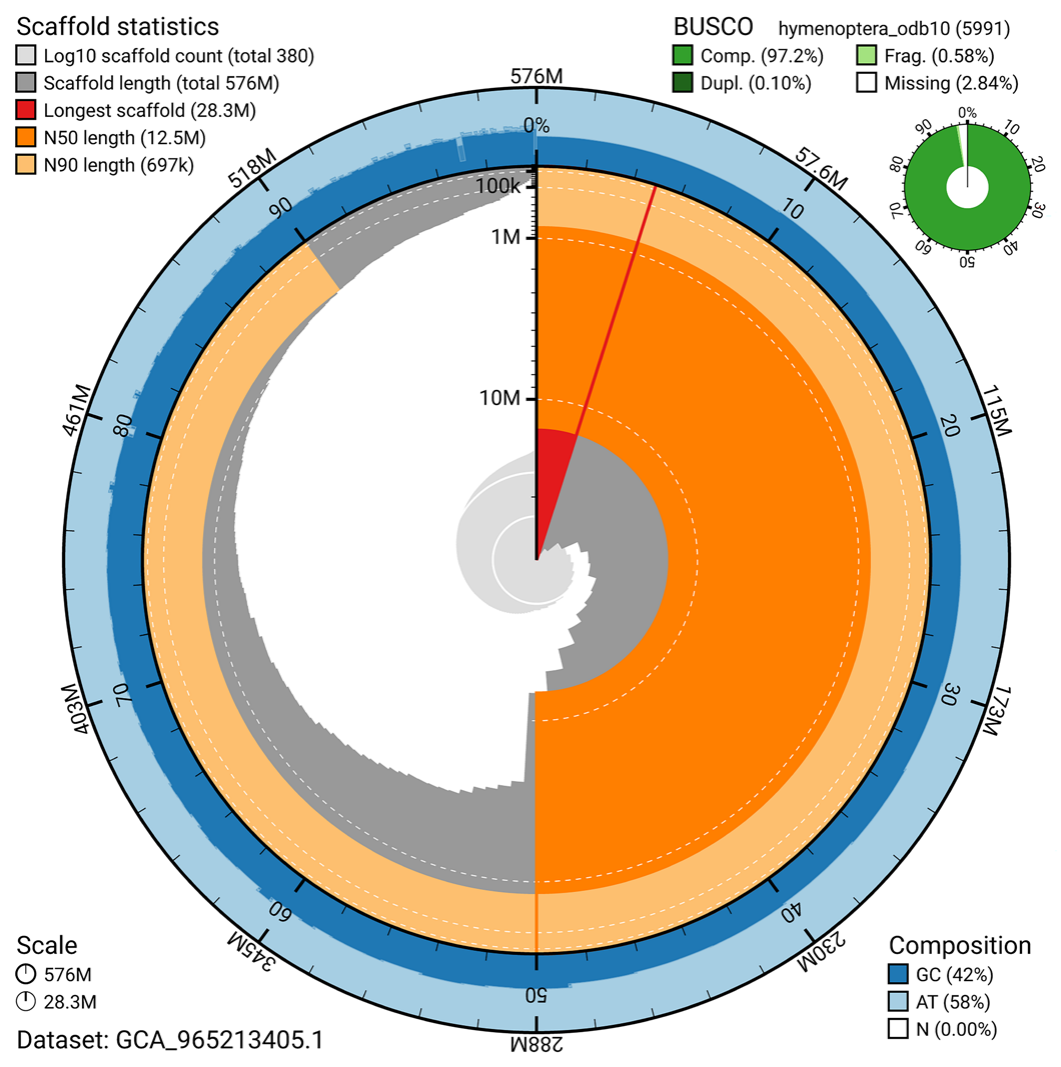
Assembly metrics for iyPhiTria1.1. The BlobToolKit snail plot provides an overview of assembly metrics and BUSCO gene completeness. The circumference represents the length of the whole genome sequence, and the main plot is divided into 1 000 bins around the circumference. The outermost blue tracks display the distribution of GC, AT, and N percentages across the bins. Scaffolds are arranged clockwise from longest to shortest and are depicted in dark grey. The longest scaffold is indicated by the red arc, and the deeper orange and pale orange arcs represent the N50 and N90 lengths. A light grey spiral at the centre shows the cumulative scaffold count on a logarithmic scale. A summary of complete, fragmented, duplicated, and missing BUSCO genes in the hymenoptera_odb10 set is presented at the top right. An interactive version of this figure can be accessed on the BlobToolKit viewer.

**Figure 6.**
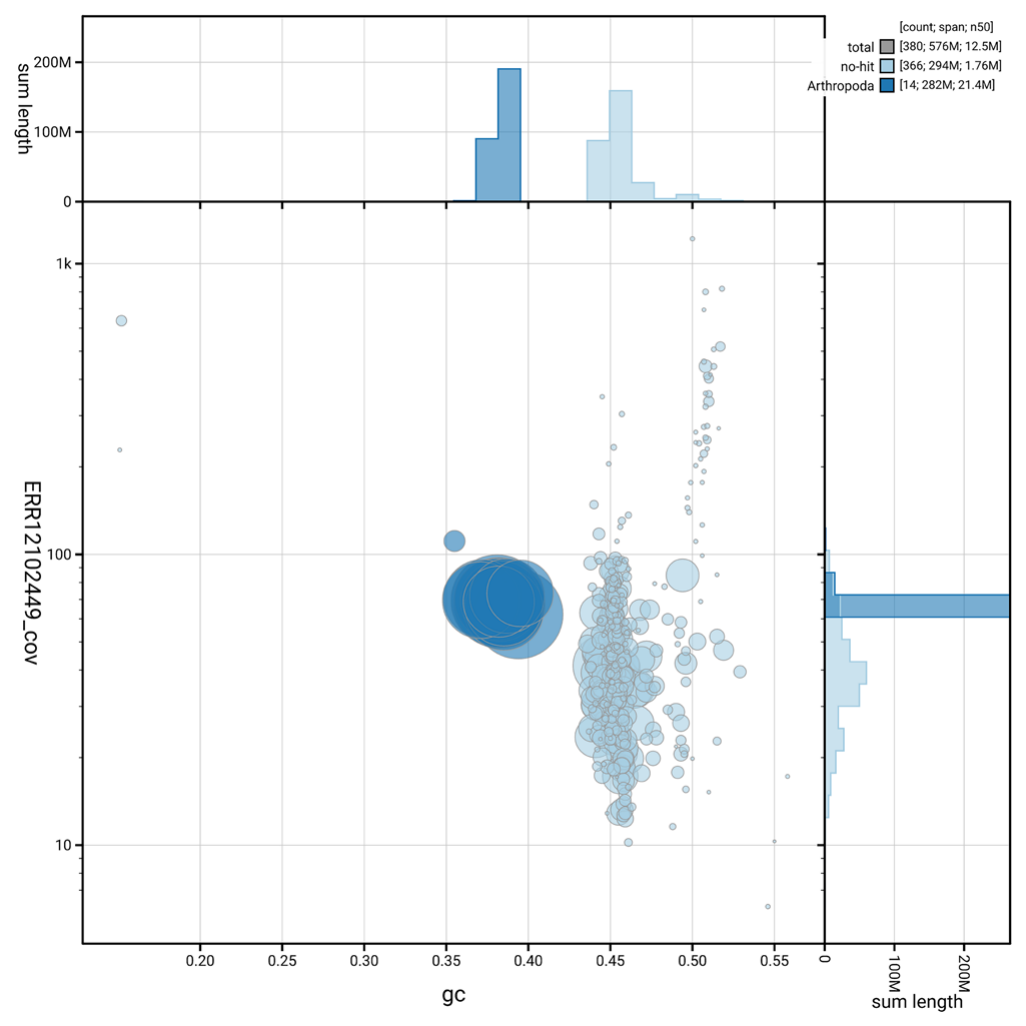
BlobToolKit GC-coverage plot for iyPhiTria1.1. Blob plot showing sequence coverage (vertical axis) and GC content (horizontal axis). The circles represent scaffolds, with the size proportional to scaffold length and the colour representing phylum membership. The histograms along the axes display the total length of sequences distributed across different levels of coverage and GC content. An interactive version of this figure is available on the BlobToolKit viewer.

BUSCO v.5.7.1 analysis using the hymenoptera_odb10 reference set ( *n* = 5 991) identified 97.2% of the expected gene set (single = 97.1%, duplicated = 0.1%). The snail plot in Figure 5 summarises the scaffold length distribution and other assembly statistics for the primary assembly. The blob plot in Figure 6 shows the distribution of scaffolds by GC proportion and coverage.

Table 4 lists the assembly metric benchmarks adapted from Rhie *et al.* (2021) the Earth BioGenome Project Report on Assembly Standards September 2024. The EBP metric, calculated for the primary assembly, is **6.7.Q65**.

**Table 4.** Earth Biogenome Project summary metrics for the *Philanthus triangulum* assembly.

Measure	Value	Benchmark
EBP summary (primary)	6.7.Q65	6.C.Q40
Contig N50 length	2.85 Mb	≥ 1 Mb
Scaffold N50 length	12.49 Mb	= chromosome N50
Consensus quality (QV)	Primary: 65.2; alternate: 65.1; combined: 65.2	≥ 40
*k*-mer completeness	Primary: 83.31%; alternate: 74.39%; combined: 99.39%	≥ 95%
BUSCO	C:97.2% [S:97.1%; D:0.1%]; F:0.6%; M:2.3%; n:5 991	S > 90%; D < 5%
Percentage of assembly assigned to chromosomes	49.26%	≥ 90%

### Wellcome Sanger Institute – Legal and Governance

The materials that have contributed to this genome note have been supplied by a Darwin Tree of Life Partner. The submission of materials by a Darwin Tree of Life Partner is subject to the **‘Darwin Tree of Life Project Sampling Code of Practice’**, which can be found in full on the Darwin Tree of Life website. By agreeing with and signing up to the Sampling Code of Practice, the Darwin Tree of Life Partner agrees they will meet the legal and ethical requirements and standards set out within this document in respect of all samples acquired for, and supplied to, the Darwin Tree of Life Project. Further, the Wellcome Sanger Institute employs a process whereby due diligence is carried out proportionate to the nature of the materials themselves, and the circumstances under which they have been/are to be collected and provided for use. The purpose of this is to address and mitigate any potential legal and/or ethical implications of receipt and use of the materials as part of the research project, and to ensure that in doing so we align with best practice wherever possible. The overarching areas of consideration are:

- Ethical review of provenance and sourcing of the material
- Legality of collection, transfer and use (national and international)

Each transfer of samples is further undertaken according to a Research Collaboration Agreement or Material Transfer Agreement entered into by the Darwin Tree of Life Partner, Genome Research Limited (operating as the Wellcome Sanger Institute), and in some circumstances, other Darwin Tree of Life collaborators.

## Data Availability

European Nucleotide Archive: Philanthus triangulum (European beewolf). Accession number PRJEB66736. The genome sequence is released openly for reuse. The *Philanthus triangulum* genome sequencing initiative is part of the Darwin Tree of Life Project (PRJEB40665) and the Sanger Institute Tree of Life Programme (PRJEB43745). All raw sequence data and the assembly have been deposited in INSDC databases. The genome will be annotated using available RNA-Seq data and presented through the Ensembl pipeline at the European Bioinformatics Institute. Raw data and assembly accession identifiers are reported in Table 1 and Table 2. Production code used in genome assembly at the WSI Tree of Life is available at https://github.com/sanger-tol. Table 5 lists software versions used in this study.

## Author information

Contributors are listed at the following links:

- Members of the Natural History Museum Genome Acquisition Lab
- Members of the Darwin Tree of Life Barcoding collective
- Members of the Wellcome Sanger Institute Tree of Life Management, Samples and Laboratory team
- Members of Wellcome Sanger Institute Scientific Operations – Sequencing Operations
- Members of the Wellcome Sanger Institute Tree of Life Core Informatics team
- Members of the Tree of Life Core Informatics collective
- Members of the Darwin Tree of Life Consortium
