# Supplementary material for: The genome sequence of the Bee Wolf, Philanthus triangulum (Fabricius, 1775) (Hymenoptera: Crabronidae)
Source: Wellcome Open Res. 2025 Oct 3;10:543. [Version 1] doi: 10.12688/wellcomeopenres.24951.1 (PMC12596551; doi:10.12688/wellcomeopenres.24951.1)
Supplement: Peer review history

# Reviewer report: Reviewer response for version 1

**DOI:** 10.21956/wellcomeopenres.27484.r135319

**Published:** 2025-11-08

**Authors:** Natalia Araujo

**Affiliations:** 1 Universite Libre de Bruxelles, Brussels, Belgium

The authors report the nuclear and mitochondrial genome assemblies of the Bee Wolf ( *Philanthus triangulum*). The final nuclear assembly size is approximately 575 Mb, with an N50 of 12.49 Mb and a BUSCO completeness score of 97.2%. Despite sufficient Hi-C data coverage being generated, only about half of the assembly was incorporated into pseudochromosome-level scaffolds. I do not consider this a methodological shortcoming, but rather a reflection of the intrinsic genomic characteristics of this species. The analyses clearly indicate that the genome is highly repetitive. In Figure 6, it is evident that roughly half of the genome consists of GC-rich scaffolds lacking BLAST matches to other species.

 As this represents the first genome assembly for the genus *Philanthus*, it constitutes a valuable contribution to the understanding of Hymenoptera genomics. The authors employed the standard Tree of Life pipeline, the methods are adequately described, and the data are openly accessible.

 Minor comments and suggestions:

1. When describing the species distribution, the authors state:
*“…the wasp is now commonly found throughout the southern counties, extending to the northern midlands…”*
  It is unclear which geographic regions are meant by “southern counties” and “northern midlands”. It is not clear to me which locations these “southern counties” and “northern midlands” correspond to.
2. No quality information is provided for the mitochondrial genome (e.g., completeness level, number of genes annotated, or coverage statistics). Including these details would strengthen the manuscript.
3. The authors mention that two alternative haplotypes were assembled, but only one assembly accession is provided. Does this mean that the scaffolds corresponding to the alternative haplotype are included in the same FASTA file as the primary assembly? This is not clear. Typically, separate FASTA files are submitted for each haplotype assembly.

Are sufficient details of methods and materials provided to allow replication by others?

Yes

Is the rationale for creating the dataset(s) clearly described?

Yes

Are the datasets clearly presented in a useable and accessible format?

Yes

Are the protocols appropriate and is the work technically sound?

Yes

Reviewer Expertise:

Genomics, Bioinformatics

I confirm that I have read this submission and believe that I have an appropriate level of expertise to confirm that it is of an acceptable scientific standard.

# Reviewer report: Reviewer response for version 1

**DOI:** 10.21956/wellcomeopenres.27484.r135326

**Published:** 2025-10-14

**Authors:** Christopher Wyatt

**Affiliations:** 1 University College London, London, England, UK

This is an important genome of an at-risk species in the UK, which now has a high quality genome which can be used by the scientific community. 

 The authors clearly define the main features of this species and the genome methodology is consistent with existing genome note publications. Despite not resolving all the contigs, the chromosomes appear largely complete with evidence from the HiC maps and BUSCO completion at 97.2%. 

 Very minor comments.

 BUSCO has a stable release v6 which could be useful to change to.

 Is it ok to use "bee wolf" and "beewolf"?, as maybe in the title it uses the old way of writing it, and I think "beewolf" as a single word is the more modern format, but I could be wrong.

Are sufficient details of methods and materials provided to allow replication by others?

Yes

Is the rationale for creating the dataset(s) clearly described?

Yes

Are the datasets clearly presented in a useable and accessible format?

Yes

Are the protocols appropriate and is the work technically sound?

Yes

Reviewer Expertise:

Genomics, Transcriptomics

I confirm that I have read this submission and believe that I have an appropriate level of expertise to confirm that it is of an acceptable scientific standard.
